# Advancements in photodynamic therapy of esophageal cancer

**DOI:** 10.3389/fonc.2022.1024576

**Published:** 2022-11-17

**Authors:** Dorota Bartusik-Aebisher, Michał Osuchowski, Marta Adamczyk, Joanna Stopa, Grzegorz Cieślar, Aleksandra Kawczyk-Krupka, David Aebisher

**Affiliations:** ^1^ Department of Biochemistry and General Chemistry, Medical College of The University of Rzeszów, Rzeszów, Poland; ^2^ Medical College of The University of Rzeszów, Rzeszów, Poland; ^3^ Medical Faculty, Medical University of Warsaw, Warsaw, Poland; ^4^ Department of Internal Medicine, Angiology, and Physical Medicine, Center for Laser Diagnostics and Therapy, Medical University of Silesia in Katowice, Bytom, Poland; ^5^ Department of Photomedicine and Physical Chemistry, Medical College of The University of Rzeszów, Rzeszów, Poland

**Keywords:** photodynamic therapy, esophageal cancer, Barrett’s esophagus, high grade dysplasia, 5-ALA, porfimer sodium, temoporfin, talaporfin

## Abstract

The poor prognosis of patients with esophageal cancer leads to the constant search for new ways of treatment of this disease. One of the methods used in high-grade dysplasia, superficial invasive carcinoma, and sometimes palliative care is photodynamic therapy (PDT). This method has come a long way from the first experimental studies to registration in the treatment of esophageal cancer and is constantly being improved and refined. This review describes esophageal cancer, current treatment methods, the introduction to PDT, the photosensitizers (PSs) used in esophageal carcinoma PDT, PDT in squamous cell carcinoma (SCC) of the esophagus, and PDT in invasive adenocarcinoma of the esophagus. For this review, research and review articles from PubMed and Web of Science databases were used. The keywords used were “photodynamic therapy in esophageal cancer” in the years 2000–2020. The total number of papers returned was 1,000. After the review was divided into topic blocks and the searched publications were analyzed, 117 articles were selected.

## Esophageal cancer

Esophageal cancer is diagnosed in advanced stages. Esophageal cancer needs improved detection and prediction methods prior to cancer treatment. Esophageal cancer originates in the epithelial cells that line the esophagus. The treatments for esophageal cancer depend on its etiology. Malignant neoplasms include squamous cell carcinoma and adenocarcinoma; 95% of all esophageal malignancies are squamous cell carcinomas or adenocarcinomas but other types of cancer, including other carcinomas, melanomas, leiomyosarcomas, carcinoids, and lymphomas, have also been reported (1, 2). Esophageal cancer is the eighth most common form of cancer worldwide. The increased risk factors for developing esophageal cancer include smoking (3, 4), the consumption of high-percentage alcohol (5), obesity (6), long-term inflammation of the esophagus mucosa (7), achalasia (8), atrophic inflammation of the tongue and esophagus (9), Barrett’s esophagus (10, 11), burns of the esophagus with chemicals (12), occupational exposure to vulcanization products (13), asbestos and metal dust (14), dietary factors (11), vitamin deficiencies (15) and trace elements, and frequent consumption of hot and pickled foods (16).

In patients with esophageal carcinoma *in situ* and lesions limited to the mucosa, local endoscopic resection may be used. Neoplastic lesions of a more advanced stage (beyond the mucosa) are indications for surgery. In some cases, preoperative chemoradiotherapy is used. In patients who are not eligible for surgery, radical chemoradiotherapy is recommended. The goal of palliative treatment in inoperable or disseminated esophageal cancer is to provide natural nutrition, slow disease progression, and improve quality of life. In order to restore the esophagus, prosthesis, laser treatment of the esophagus, or intra-esophageal brachytherapy can be used. The effectiveness of palliative chemotherapy is greater in patients with adenocarcinoma of the esophagus, but it is not associated with a significant increase in the overall survival time of patients. In some cases of advanced esophageal cancer, a nutritional gastrostomy or jejunostomy may be necessary. The reasons for its rapidly increasing incidence include the rising prevalence of gastroesophageal reflux and obesity combined with the decreasing prevalence of *Helicobacter pylori* infection (17).

For mucosal cancer, endoscopic mucosal resection and endoscopic submucosal dissection are standard, while for locally advanced cancer, esophagectomy remains the mainstay. The three most common techniques for thoracic esophagectomy are transhiatal approach, the Ivor–Lewis esophagectomy (right thoracotomy and laparotomy), and the McKeown technique (right thoracotomy followed by laparotomy and neck incision with cervical anastomosis). Surgery for carcinoma of the cervical esophagus requires an extensive procedure with laryngectomy in many cases. When the tumor is more advanced, neoadjuvant chemotherapy or neoadjuvant chemoradiotherapy is added. Neoadjuvant concurrent chemoradiotherapy (CCRT) is a strategy to decrease tumor size. However, CCRT may enhance toxicity levels and possibly cause a delay in surgery for patients who respond poorly to CCRT (18). The theoretical advantages of adding chemotherapy to the treatment of esophageal cancer are potential tumor down-staging prior to surgery, as well as targeting micrometastases and, thus, decreasing the risk of distant metastasis. Cisplatin- and 5-fluorouracil-based regimens are used worldwide. Chemoradiotherapy is the standard for unresectable esophageal cancer and could also be considered an option for resectable tumors. For patients who are medically or technically inoperable, concurrent chemoradiotherapy should be the standard of care. Although neoadjuvant chemoradiotherapy followed by surgery or salvage surgery after definitive chemoradiotherapy is a practical treatment, judicious patient selection is crucial. It is important to have a thorough understanding of these therapeutic modalities to assist in this endeavor. Despite advances in surgical techniques and optimization of chemoradiotherapy regimens, overall survival benefits have been incremental at best. Esophageal cancer requires a concerted multidisciplinary approach, perhaps more so than any other tumor type given the integral role played by the esophagus in maintaining calorific intake and the propensity for early spread through the lymphatics (19–21).

Early cancer detection is the most important, and numerous imaging and diagnostic methods are utilized for this purpose, including computed tomography (CT) (22), magnetic resonance imaging (MRI) (23), positron emission tomography (PET) (24), and endoscopic procedures (25) and especially gastroscopy (26).

In this review, the authors searched through the available literature and analyzed the available photosensitizers, methods of carrying out the procedure, the effects of PDT treatment of esophageal cancer, and concepts for the future development of new therapy.

**Figure 1:**
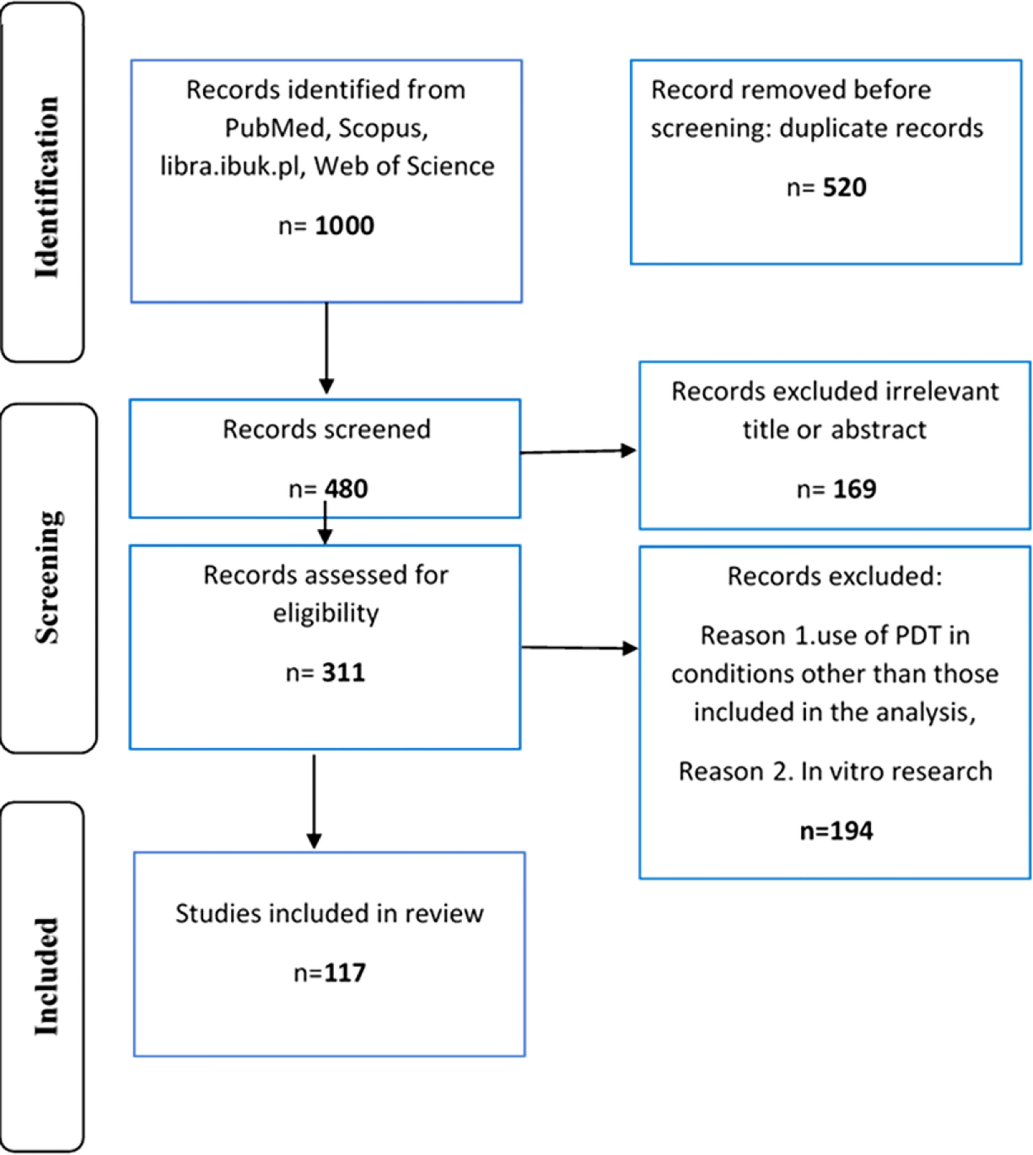
Diagram illustrating the procedure for analyzing the source articles.

**Figure 1** shows a diagram illustrating the procedure for analyzing the source articles.

## Treatment methods

Surgery is an important component of treatment for esophageal cancer (27).

However, surgery alone presents poor overall survival rates; therefore, combined modality therapy has been introduced for the treatment of esophageal cancer (28). Randomized trials have proven that preoperative chemoradiation (CRT) and perioperative chemotherapy significantly improved survival in patients with respectable esophageal and gastroesophageal junction cancers (29–32).

If due to clinical indications a patient with locally advanced or metastatic cancer cannot be treated surgically, chemotherapy should be considered. Cisplatin has proven to be an efficient chemotherapeutic agent, with a single-agent response rate of approximately 20% or even higher (33). Other anti-cancer drugs including irinotecan (34, 35), docetaxel (36), paclitaxel (37, 38), etoposide (39), and more recently gemcitabine (40, 41) cisplatin plus paclitaxel or docetaxel, with or without 5-fluorouracil (5-FU) have also demonstrated activity in patients with locally advanced or metastatic disease (42–44). Palliative care patients and those with esophageal obstruction may also benefit from photodynamic therapy (PDT) (45). The chosen treatment method must be personalized to the individual needs of a particular patient (46).

## Photodynamic therapy

**Figure 2:**
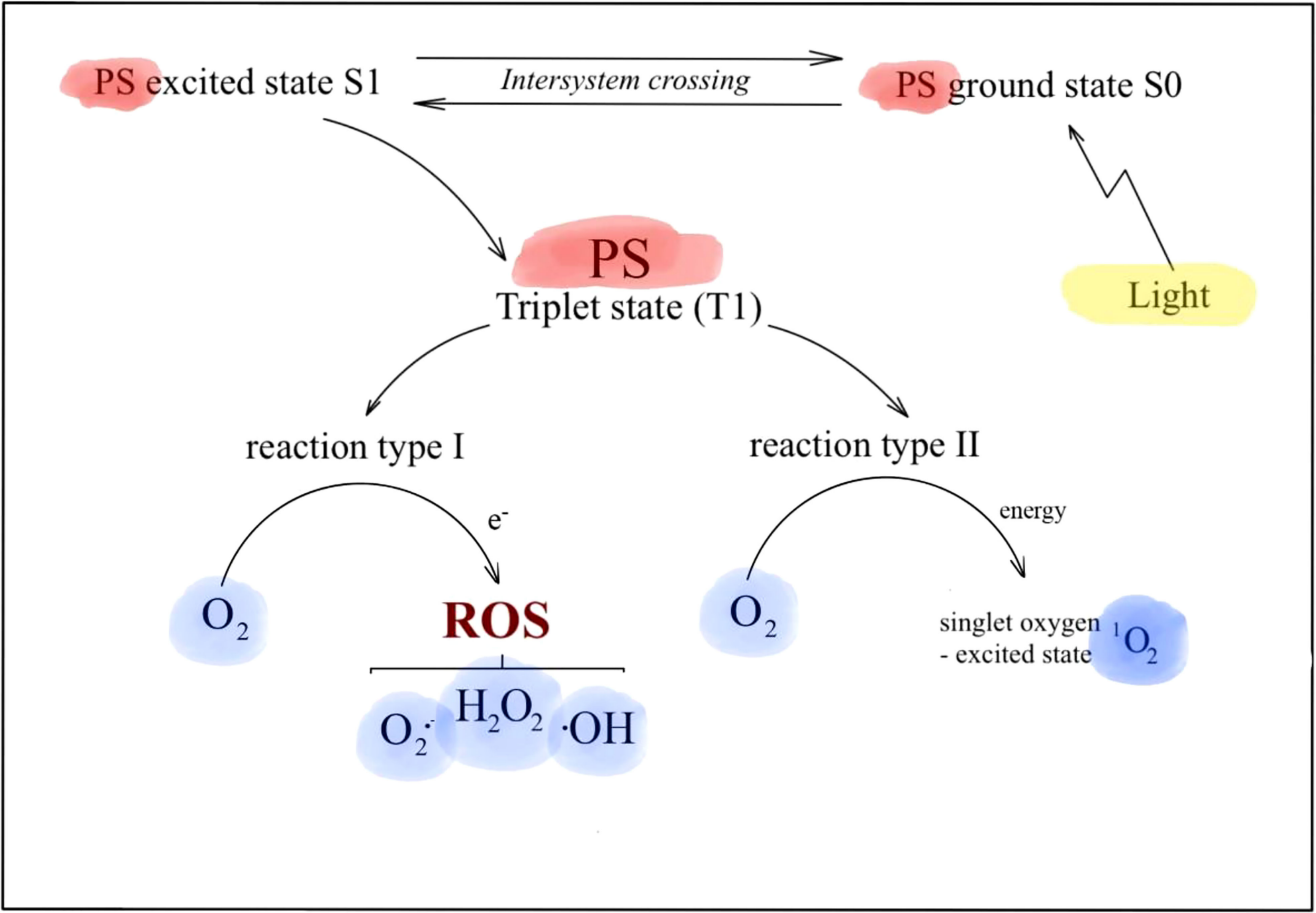
The mechanism of photodynamic therapy (PDT).

PDT is a treatment when the tumor site is irradiated with light of an appropriate wavelength in the presence of a photosensitizer (PS) (**Figure 1**). The main mechanism of PDT is based on the generation of reactive oxygen species (ROS), which are lethal to cancer tissues by damaging them directly (necrosis) and inducing apoptosis (47). Additionally, it has an indirect effect by modifying tumor vascularization and stimulating the immune response of the patient (48). PDT acts selectively, only at the site where the light is provided, thus accounting for fewer adverse effects than systemic treatment. **Figure 2** presents the mechanism of PDT and reactive species generation.

The side effects include phototoxicity due to PSs accumulating in healthy tissues, which is why patients should avoid sunlight during treatment. The downside is also its limited use. PDT is not an efficient treatment method for patients with lymph nodes or distant metastases (49).

## The photosensitizers used in esophageal carcinoma photodynamic therapy

Photosensitizers (PSs) are molecules that build up in cancerous cells and less intensively in healthy cells. The effectiveness of PDT (**Supplementary Figure 1**) is based on PSs used; therefore, a large number of clinical studies are aimed at the synthesis and optimization of physicochemical photoactive (PT) compounds (50). There are several characteristics of an optimal PS: availability of pure chemical substance, long-wavelength absorbing (wavelengths from 600 to 800 nm), strong photocytotoxicity, selectivity in accumulation in target cells, not having phototoxic effects in normal tissues, the absorption bands of the photosensitizer different from absorption of endogenous dyes, e.g., melanin or hemoglobin, the smallest possible number of side effects, easy and rapid excretion from the body, ease of administration through various routes, low cost, and simple synthesis (51–54).

There are various ways of PS classifications, such as classification due to chemical structure: porphyrins, chlorins, bacteriochlorins, and phthalocyanines with their derivatives (54). The first-generation PSs are porphyrin/hematoporphyrin and their derivatives (hematoporphyrin derivatives (HpD)). The second-generation PSs have various structures including porphyrins, chlorophyll derivatives, and dyes. Third-generation PSs contain first- and second-generation PSs conjugated to various modifiers such as antibodies and nanoparticles (55, 56). Among all those molecules, the most common and clinically approved in esophageal diseases are porfimer sodium (Photofrin), mTHPC/temoporfin (Foscan), talaporfin sodium (Laserphyrin), and 5-aminolevulinic acid (Alabel) (57). Porfimer sodium, mTHPC, and 5-ALA are activated by similar red light energy (630, 652, and 635 nm, respectively) and produce a depth of mucosal necrosis varying from 6 to 7 mm for Photofrin, 5 to 10 mm for Foscan, and 2 mm for 5-ALA (58–63). Talaporfin sodium is expected to reach deeper layers including the muscularis propria because the excitation wavelength of the diode laser used is longer than in the excimer dye laser used in other PSs (**Supplementary Figure 2**). The general instrumental setup is presented in (**Supplementary Figures 3, 5**).

### Porfimer sodium

Porfimer sodium (**Supplementary Figure 4**), a first-generation PS, is the most widely used and investigated PS in esophageal PDT. After injection into a vein, the drug is removed from most tissues within 40–72 h. It remains significantly longer in tumors, skin, and organs of the reticuloendothelial system. It is excited with 630-nm light, which initiates a photodynamic reaction leading to the destruction of abnormal cells (64–66). Many clinical trials using porfimer sodium were carried out, and porfimer sodium is currently approved for use in PDT worldwide.

Lightdale et al. compared the PDT with porfimer sodium with thermal ablation therapy with Nd : YAG laser in the palliative treatment of esophageal cancer (67). The result of the therapy with PDT was the eradication of the segment of Barrett’s esophagus (BE). This finding led to many clinical trials (randomized, follow-up, and retrospective) testing the effectiveness of porfimer sodium PDT in the treatment of dysplastic BE (68–72). The study findings resulted in the approval of porfimer sodium PDT for the treatment of high-grade dysplasia associated with Barrett’s metaplasia (BE-HGD) and superficial esophageal adenocarcinoma (73–76). Current recommendations for porfimer sodium PDT (manufacturer/Food and Drug Administration (FDA)) for BE and esophageal cancer lesions are as follows: ablation of high-grade dysplasia in the BE in patients not undergoing surgery, cancer lesions smaller than half of the circumference of the lumen and 2 cm in diameter that are limited to the submucosal layer in depth and lesions (which are difficult to remove with endoscopic resection), and also the palliative treatment of patients with completely or partially obstructing esophageal cancer (**Supplementary Figure 5**). Other applications include the ablation of non-dysplastic Barrett’s mucosa (77).

### 5-ALA

5-ALA (**Supplementary Figure 6**) is a second-generation PS. It is a pro-drug that stimulates the endogenous production of protoporphyrin IX, mostly within the gut mucosa (78, 79). There are some important benefits of using 5-ALA in gastrointestinal tract diseases (80–82). It preferentially accumulates in tumors as compared with normal cells. An important advantage of using 5-ALA PDT is the short time period of photosensitivity after the procedure, lasting only 24 to 48 h (83–85). It targets the superficial mucosal layer and therefore rarely induces the development of strictures (81).

In a study by Tan et al., 5-ALA-PDT presented insufficient tumor selectivity in the treatment of esophageal adenocarcinoma, and thus, only carcinoma *in situ* could be eradicated (86). However, it could relieve dysphagia in patients with strictures (87). Another disadvantage of 5-ALA PDT is a high recurrence rate in patients with early cancer (65). So far, the 5-ALA PDT procedure was applied mostly in Europe, Scandinavia, and the United States for the treatment of patients with BE-HGD (88).

### Temoporfin (mTHPC)

mTHPC (**Supplementary Figure 7**) is a second-generation PS and is associated with photosensitivity lasting for 2 to 3 weeks after administration. In gastroenterology, mTHPC has been used intravenously at a dose of 0.15 mg/kg with 652-nm light activation (89). There were only a few clinical studies, mainly in Europe, evaluating the role of mTHPC in the treatment of BE-HGD and early esophageal cancer (90, 91). Gossner et al. used mTHPC as a complementary therapy in a small number of patients with BE-HGD who had failed previous treatment with 5-ALA PDT (92). In 2002, Javaid et al. treated patients with BE-HGD using mTHPC with an argon-pump dye laser light of 652 nm and a xenon arc lamp with equivalent results, demonstrating that efficient photosensitizers may not require high-power laser light sources for effective activation (93). Some studies report initial positive results in using mTHPC in BE-HGD and superficial esophageal cancer with green light (at 514 nm) (89–93), but none of the patients had successful disease eradication or reached a long-term remission (90).

### Talaporfin sodium

Talaporfin sodium (**Supplementary Figure 8**), a second-generation PS utilized in Japan (Laserphyrin for injection; Meiji Seika Pharma, Tokyo, Japan), has fast skin clearance and is associated with photosensitivity lasting for only 2 weeks (94, 95). Talaporfin sodium can reach deeper layers in the muscularis propria (39). The talaporfin sodium used for the PDT procedure consists of i.v. administration of 40 mg/m^2^ dose of the PSs followed by laser illumination at a 664-nm wavelength 4–6 h after administration (45). The first clinical trials with talaporfin sodium assessed the tissue damage of a normal esophagus caused by photo-activation in a living canine model (78). After that, phase I and II clinical trials were planned and carried out by Yano et al. to assess the usefulness of using talaporfin sodium PDT for salvage treatment in esophageal cancer for local failures after CRT (96, 97). In 2019 and 2020, Minamide et al. and Ishida et al. respectively further confirmed that talaporfin sodium PDT is effective in patients who did not benefit from chemoradiotherapy or radiotherapy for esophageal cancer (98, 99).

### 2-[1-Hexyloxyethyl]-2-devinyl pyropheophorbide-a

Nava et al. studied 2-[1-hexyloxyethyl]-2-devinyl pyropheophorbide-a (HPPH)-PDT (**Supplementary Figure 9**) for precancerous lesions in BE. Patients treated with HPPH showed less photosensitivity than those treated with porfimer sodium. HPPH doses ranged from 3 to 6 mg/m^2^, and lesions were irritated with one endoscopic exposure to 150, 175, or 200 J/cm of light with a wavelength of 665 nm. At a 1-year follow-up, 72% of patients had complete remission (no dysplasia or cancer present). Side effects included mild-to-moderate chest pain requiring symptomatic treatment in most patients and grade 3 and 4 adverse events in 16.6% of patients including esophageal strictures. The authors concluded that further clinical studies are required to establish the usefulness of HPPH-PDT in esophageal carcinoma (100).

## Photodynamic therapy in squamous cell carcinoma of the esophagus

PDT was used in patients with early-stage esophageal squamous cell carcinoma (SCC) with curative intent. Seven patients with SCC or HGD were included in the experiment, and none of them had lymph node metastases. The team injected porfimer sodium intravenously (2 mg/kg) and exposed the tumor site to a laser with a wavelength of 630 nm. In some of the cases, a second irradiation of the lesion was performed. All treated lesions were eradicated. Follow-up (range 4–51 months) did not show a recurrence in any of the patients. There were no adverse effects after the procedure (50).

Yano et al. described 13 patients with initial treatment failure: nine patients with remaining tumors after CRT and four patients with tumor recurrence. Inclusion criteria included no metastases in lymph nodes, T1 or T2 stage, and contraindications to surgical treatment. Eight patients (62%) had complete remission after treatment, and at a 12-month follow-up, nine were still alive and six were still disease-free. The overall survival rate after salvage PDT after 1 year was 68.4%. During the PDT treatments, six patients experienced significant complications: esophagotracheal fistula (1), stenosis (3), skin phototoxicity (1), and radiation-induced pleural effusion (1). The authors expressed hope that PDT could be used as a treatment with curative intent (97).

Takana et al. assessed 52 patients with esophagus cancer who underwent PDT from 1999 to 2007 in a retrospective study. Fourteen patients had a different type of therapy prior to PDT, and 31 patients received PDT only. Photosensitizer was administrated 48 h prior to light irradiation (excimer laser, 75 J/cm^2^). Complete remission was obtained in 33 patients (87%): 25 patients after the first PDT and 8 patients after more than one course of PDT. Four patients had recurrence after 12 months: two of them were successfully treated with another PDT course, and two developed lung or lymph node metastases. Common complications were chest pain and fever >38°C, all managed with non-steroidal anti-inflammatory drugs. Cutaneous phototoxicity was observed in 6 patients (16%) (101).

In another study, Yano et al. treated 25 patients who previously underwent CRT with PDT using talaporfin sodium. This was meant to be a salvage therapy for patients with esophageal cancer recurrence (14 patients) or residual lesions (11 patients). Complete remission was observed in 76% (19/25) of patients. The median follow-up was 48 months. At that time, only 11 patients from the complete remission group were disease-free. The commonly reported adverse effects of PDT were chest pain (61%), pharyngeal pain (17%), dysphagia (39%), fever (48%), and photosensitivity (32%). There was one treatment-related death: the patient developed severe gastrointestinal hemorrhage (102).

In 2012, Yano et al. achieved a complete response after PDT of esophageal SCC in five of nine patients (55.6%). In this study, they found optimal laser irradiation fluence rate for PDT using talaporfin sodium and diode laser (100 J/cm^2^) and achieved no dermatological adverse effects (103).

Lindenmann et al. presented a retrospective study about an individualized approach to palliative procedures in esophageal cancer that included PDT. They evaluated 248 patients excluded from surgery. PDT with hematoporphyrin was performed in 171 cases (first treatment in 118 cases). The median survival rate was 50.9 months if PDT was the initial treatment and 17.3 months if other methods were used first. The mean survival time for all patients was 34 months. The side effects of PDT included esophageal tumor perforation within 5 days from PDT (8.8%) and tumor necrosis-associated hemorrhage (7.6%). PDT as an initial endoluminal treatment improved and prolonged the survival rate of patients without massive invasion of the mediastinum, trachea, bronchial tree, or great vessels (104).

In 2017, Yano et al. presented results of PDT in patients with local failure of CRT (21 patients) or radiotherapy (5 patients) in esophageal SCC carcinoma. There were 26 patients with 28 lesions qualified for the study. Lesions were confirmed as T1b (21) and T2 (7). Twenty-three patients with 25 lesions (88.5%) had local complete remission (L-CR). In lesions staged as T1, L-CR was 100%; in lesions staged as T2, it was 57.1%. There was no skin phototoxicity observed; 53.8% of patients suffered from esophageal pain and 30.8% from fever. The median follow-up of 8.4 months showed no death from esophageal progression, but two of three patients without L-CR developed progression, and one patient from the L-CR group suffered from recurrence after 14 months. Three patients developed lymph nodes or distal metastases (105).

## Photodynamic therapy in Barrett’s esophagus

Barrett’s esophagus is a premalignant disease that predisposes to the development of esophageal adenocarcinoma.

PDT is one of the longest-used ablation techniques in the treatment of BE. The first studies concentrated on the use of porfimer sodium in Barrett’s disease and early esophageal cancer (106). The results of these clinical trials led to the approval of PDT in the United States, Europe, and Japan, which allowed for the expansion of research. There have been many subsequent reports proving the high effectiveness of photodynamic therapy.

In the clinical trial of Overholt et al., patients with BE and HGD were divided into two groups: one using porfimer sodium PDT with concomitant proton pump inhibitor (PPI) therapy and one with PPI therapy alone. The 5-year follow-up showed that porfimer sodium PDT was significantly more effective than omeprazole, with the elimination of HGD in 77% and 39% (p < 0.0001). The second endpoint assessed was the progression to adenocarcinoma, which was 15% for the first group and 29% for omeprazole (p = 0.027), with a significantly longer progression time for the first group (p = 0.004) (73).

Equally favorable effects were achieved in a study that assessed the effects of PDT using 5-ALA in BE with HGD (group A) and in superficial esophageal cancer (group B). Of the patients, 97% in group A and 100% in group B achieved complete remission with a mean follow-up of 37 months. Local recurrence was observed in one patient in group A and 10 patients in group B. The estimated 5-year survival was 97% in group A and 80% in group B (107).

In a study comparing the effects of PDT with 5-ALA and Photofrin in patients with BE and HGD, complete dysplasia regression (CR-HGD) was achieved in 47% and 40% of cases, respectively. Esophageal stricture and photosensitivity were statistically more common in patients treated with porfimer sodium PDT (33% *vs.* 9% and 43% *vs.* 6%, p = 0.05). The study showed a better risk profile and better outcomes with BE lengths ≤ 6 cm using 5-ALA PDT. With the longer BE segment, no statistically significant difference was observed with the use of both methods (108).

There were only a few clinical studies, mainly in Europe, evaluating the role of mTHPC in BE. Gossner et al. used mTHPC PDT as salvage therapy in a small number of patients with BE-HGD who had failed previous treatment with 5-ALA PDT (109). Other studies included the treatment of both BE with high-grade and low-grade dysplasia (102, 103). The trials demonstrated that mTHPC-PDT is useful in BE PDT, but further studies are needed to establish its exact effectiveness.

In the next study, the efficacy of porfimer sodium PDT and radiofrequency ablation (RFA) was compared. The percentage of complete histopathological remissions of BE was 54.5% for PDT and 88.7% for RFA. There was one case of perforation in the PDT group, with no similar complications in the RFA group. However, the limitation of this study was the lack of randomization and the higher stage of the disease in patients treated with PDT (110).

## Photodynamic therapy in invasive adenocarcinoma of the esophagus

In Japan, PDT for esophageal carcinoma was approved for patients with superficial cancer or in case of local failure after CRT. Tan et al. described a study of 12 patients, aged 55–88, with esophageal adenocarcinoma arising from Barrett’s metaplasia. 5-ALA was chosen as the PS due to limited side effects and preferential accumulation in the mucosa and mucosal tumor. 5-ALA was given orally in the dose of 60 and 75 mg/kg body weight and irritated using laser light (630 nm) delivered *via* a cylindrical diffuser 4–6 h after the first dose of PSs. After PDT, the mucosa was examined, and histology showed fibrinoid necrosis. One patient with carcinoma *in situin-situ* had the tumor eradicated after one treatment with no recurrence at 28 months. Another patient with a small T1 tumor required four PDT treatments and had no evidence of recurrence after 36 months. The tumor size in the other, more advanced cases was not significantly reduced (86).

A study by Kashtan et al. had similar results. 5-ALA PDT did not prove to be efficient in the treatment of esophageal adenocarcinoma, due to low selectivity for tumor mucosa and eradication achieved only in preinvasive carcinomas (87).

Another PS used in PDT of esophageal adenocarcinoma was porfimer sodium (Photofrin). It has proven long-term efficacy and durability in the treatment of BE, HGD, and superficial esophageal adenocarcinoma. However, its continued use is hindered by serious side effects including prolonged cutaneous photosensitivity (4–6 weeks) and increased stricture risk (111).

In the United States, the FDA accepted PDT as a palliative treatment for patients with symptomatic obstructive esophageal cancer (SCC and adenocarcinoma) after studies comparing PDT with thermal YAG laser for patients with neoplastic esophageal obstruction were published. A previously mentioned study by Lightdale et al. is about a multicenter randomized trial that included 218 patients with advanced esophageal cancer from 24 centers. There was no significant difference in the dysphagia score, but tumor response 1 month after treatment was better in patients who underwent PDT (32% for PDT *vs.* 20% for Nd : YAG). The esophageal perforation rate was higher in the YAG laser group (PDT, 1%, *vs.* Nd : YAG, 7%), but PDT patients experienced severe skin photosensitivity (67).

Litle et al. examined 215 patients with symptomatic or recurrent esophageal cancer. In this group, adenocarcinoma was the dominant histological type (83%). Of patients who underwent PDT, 85% reported fewer swallowing disorders (94). However, the European Society of Gastrointestinal Endoscopy (ESGE) recommends metal stents as the method of choice in the treatment of dysphagia in patients with esophageal obstruction in the course of cancer (112).

The application of PDT in esophageal adenocarcinoma on a wider scale requires a better understanding of dosimetry and tissue properties and is currently limited only to superficial changes.

## Development opportunities

Lack of oxygen in the treated tissues means no ROS and no cytotoxic effect of PDT. In 2020, to face this problem, Roque et al. introduced two osmium-based polypyridyl photosensitizers (mainly 1-4T and 2-4T complexes) that are active in hypoxia. These complexes were relatively non-toxic in the absence of a light source. Phototherapeutic indices (PIs; the ratio of dark-to-light cytotoxicity) under irradiation with red and visible light (fluence of 100 J/cm^2^ and irradiance of approximately 20 mW/cm^2^) were maintained even in hypoxia (1% O_2_), which emulates an environment present in deep tissues and solid tumors. Both compounds were studied for *in vivo* treatment. This led to the determination of a maximum tolerated dose value, which turned out to be greater than or equal to 200 mg/kg in an intraperitoneal injection. The lead complexes demonstrated low toxicity *in vitro* with high tolerance in mice and are being prepared for *in vivo* validation (113). Another way to increase the efficiency of PDT is through nanocarriers. Carriers help to deliver the drug selectively to cancer cells and to multiply its concentration in the tumors while sparing healthy tissues. Nanotherapeutics as delivery tools for drugs have the potential to improve PDT therapeutic impact and are currently being developed and tested mostly in pre-clinical trials (114). The potential role of PDT in functionalized nanomedicine is often highlighted (115, 116). Fluoroscopy-guided PDT by using nanoparticle albumin-bound paclitaxel for esophageal cancer after chemoradiotherapy is known and well-described (117).

## Photodynamic therapy instruments

An important part of the PDT research of esophageal cancer is the examination of the photosensitizer/fiber optic device flowing oxygen to optimize photosensitizer delivery to the tissues. PDT literature presents that singlet oxygen diffusion in cells is shorter than the diameter of a typical intracellular organelle. The formation of singlet oxygen at a specific biological site is extremely important to understand the properties of tumor destruction by directed and concentrated singlet oxygen. Reactive products formed by interaction with singlet oxygen give rise to the desired toxic effect. Since singlet oxygen diffusion over a distance is unlikely, we hypothesize that specific/controlled accumulation of a sensitizer in a tumor may result from cleavage from a fiber probe (118).

Visible light will be available from the fiber itself. The benefits expected from the new fiber device are improved selectivity of the photosensitizer in diseased cells and tissues, high-precision control of the production of singlet oxygen on the micro scale to lethally damage diseased tissues, and a point-source fiber-based ^1^O_2_ method that is expected to kill tumor cells inaccessible by surgical methods.

It was also reported that the cationic PS-impregnated porous Vycor glass served as a new singlet oxygen generator and more importantly serves as a heterogeneous PS solid-phase PS scaffold for use in water systems without PSs being released into the water. The described heterogeneous system was then connected to a hollow optical fiber for supplying light and oxygen for wastewater treatment and as the first PDT approximation device (119) (**Supplementary Figure 10**).

The use of PDT aims to improve the methods of cytotoxic drug delivery, especially in terms of improving therapy and searching for improved methods of monitoring and visualizing their delivery. Currently, there are several PDT treatments approved for use in clinical medicine and several clinical trials. Photofrin^®^ was the first PS approved for use in PDT in the treatment of bladder cancer, esophageal cancer, non-small cell lung cancer, esophageal cancer, and cervical cancer. Chlorin e6 (talaporfin sodium, approved for lung cancer in Japan) and Photochlor (in clinical trials in esophageal cancer, basal cell carcinoma, lung cancer, and Barrett’s esophagus) are two PSs that I am researching to increase the depth of treatment in PDT therapy. From current data, it appears that Tookad^®^ is a promising PS to find in prostate cancer clinical trials in the United States. There are also PSs approved for age-related macular degeneration (Visudine) and corneal degeneration (Levulan^®^ and Metvixia). The primary limitation of this promising methodology is the depth of action at which visible light can penetrate the tissues, which ranges from a few millimeters (blue–green light) to just over 1 cm (red light). For example, Tookad^®^, which has found application in colon cancer clinical trials, absorbs red light at 761 nm and has been reported to induce tissue necrosis to a depth of 1.3 cm. Research has also been performed to develop a PS that dips near-infrared rays that can penetrate tissues to a depth of more than 2 cm. A fiber optic-based singlet oxygen generator for targeted singlet oxygen delivery is proposed for use in photodynamic therapy and drug delivery. The heterogeneous photodynamic therapy device that uses the optical excitation of sensitizer molecules released from porous ends on hollow photonic band-gap optical fibers through which O_2_ flows is still a challenge in clinical studies (120–122).

## Author contributions

Conceptualization, DB-A, MO, MA, JS, GC, AK-K, and DA; methodology, DB-A, MO, MA, JS, GC, AK-K, and DA; software, DB-A, MO, MA, JS, GC, AK-K, and DA; validation, DB-A, MO, MA, JS, GC, AK-K, and DA; formal analysis, DB-A, MO, MA, JS, GC, AK-K, and DA; investigation, DB-A, MO, MA, JS, GC, AK-K, and DA; resources, DB-A, MO, MA, JS, GC, AK-K, and DA; data curation, writing—original draft preparation, DB-A, MO, MA, JS, GC, AK-K, and DA. All authors contributed to the article and approved the submitted version.

## Conflict of interest

The authors declare that the research was conducted in the absence of any commercial or financial relationships that could be construed as a potential conflict of interest.

## Publisher’s note

All claims expressed in this article are solely those of the authors and do not necessarily represent those of their affiliated organizations, or those of the publisher, the editors and the reviewers. Any product that may be evaluated in this article, or claim that may be made by its manufacturer, is not guaranteed or endorsed by the publisher.

## References

[1] Nagtegaal ID Odze RD Klimstra D Paradis V Rugge M Schirmacher P . WHO classification of tumours editorial board. 2019 WHO classification tumours digestive system. Histopathology (2020) 76(2):182–8. doi: 10.1111/his.13975 PMC7003895 31433515

[2] Short MW Burgers KG Fry VT . Esophageal cancer. Am Fam Physician (2017) 95(1):22–8. 28075104

[3] Oze I Matsuo K Ito H Wakai K Nagata C Mizoue T . Cigarette smoking and esophageal cancer risk: An evaluation based on a systematic review of epidemiologic evidence among the Japanese population. Japanese J Clin Oncol (2011) 42(1):63–73. doi: 10.1093/jjco/hyr170 22131340

[4] Dong J Thrift AP . Alcohol, smoking and risk of oesophago-gastric cancer. Best Pract Res Clin Gastroenterol (2017) 31(5):509–17. doi: 10.1016/j.bpg.2017.09.002 29195670

[5] Carvalho LFCS Dos Santos L Bonnier F O'Callaghan K O'Sullivan J Flint S . Can ethanol affect the cell structure? a dynamic molecular and raman spectroscopy study. Photodiagnosis Photodyn Ther (2020) 30:101675. doi: 10.1016/j.pdpdt.2020.101675 31991233

[6] Nimptsch K Steffen A Pischon T . Obesity and oesophageal cancer. Recent Results Cancer Res (2016) 208:67–80. doi: 10.1007/978-3-319-42542-9_4 27909902

[7] Siebert M Ribeiro-Parenti L Nguyen ND Hourseau M Duchêne B Humbert L . Long-term consequences of one anastomosis gastric bypass on esogastric mucosa in a preclinical rat model. Sci Rep (2020) 10(1):7393. doi: 10.1038/s41598-020-64425-2 32355175 PMC7192900

[8] Sato H Terai S Shimamura Y Tanaka S Shiwaku H Minami H . Achalasia and esophageal cancer: a large database analysis in Japan. J Gastroenterol (2021) 56(4):360–70. doi: 10.1007/s00535-021-01763-6 33538893

[9] Krenzke LR Cameron S Pritchard JC Webb DB Guo LT Shelton GD . Glossitis in an older non-corgi dog: Diagnosis and long-term follow-up. Can Vet J (2022) 63(8):825–9. PMC9281888 35919473

[10] Enzinger PC Mayer RJ . Esophageal cancer. New Engl J Med (2003) 349(23):2241–52. doi: 10.1056/nejmra035010 14657432

[11] Tay SW Li JW Fock KM . Diet and cancer of the esophagus and stomach. Curr Opin Gastroenterol (2021) 37(2):158–63. doi: 10.1097/MOG.0000000000000700 33315794

[12] Kraft R Jensen KO Jeschke MG Schinkel CW . Patient with scald burn of the esophagus. J Burn Care Res (2018) 39(3):468–70. doi: 10.1097/BCR.0000000000000595 28570308

[13] Gustavsson P Evanoff B Hogstedt C . Increased risk of esophageal cancer among workers exposed to combustion products. Arch Environ Health (1993) 48(4):243–5. doi: 10.1080/00039896.1993.9940366 8357273

[14] Sjödahl K Jansson C Bergdahl IA Adami J Boffetta P Lagergren J . Airborne exposures and risk of gastric cancer: a prospective cohort study. Int J Cancer. (2007) 120(9):2013–8. doi: 10.1002/ijc.22566 17266028

[15] Mahendra A Karishma Choudhury BK Sharma T Bansal N Bansal R . Vitamin d and gastrointestinal cancer. J Lab Physicians. (2018) 10(1):1–5. doi: 10.4103/JLP.JLP_49_17 29403195 PMC5784277

[16] Tian D Mo SJ Han LK Cheng L Huang H Hao S . Investigation of dietary factors and esophageal cancer knowledge: Comparison of rural residents in high- and low-incidence areas. Sci Rep (2018) 8(1):4914. doi: 10.1038/s41598-018-23251-3 29559669 PMC5861081

[17] Kato H Nakajima M . Treatments for esophageal cancer: a review. Gen Thorac Cardiovasc Surg (2013) 61(6):330–5. doi: 10.1007/s11748-013-0246-0 23568356

[18] Nakajima M Kato H . Treatment options for esophageal squamous cell carcinoma. Expert Opin Pharmacother. (2013) 14(10):1345–54. doi: 10.1517/14656566.2013.801454 23675862

[19] Kelly RJ . Emerging multimodality approaches to treat localized esophageal cancer. J Natl Compr Canc Netw (2019) 17(8):1009–14. doi: 10.6004/jnccn.2019.7337 31390584

[20] Huang FL Yu SJ . Esophageal cancer: Risk factors, genetic association, and treatment. Asian J Surg (2018) 41(3):210–5. doi: 10.1016/j.asjsur.2016.10.005 27986415

[21] Lagergren J Lagergren P . Recent developments in esophageal adenocarcinoma. CA Cancer J Clin (2013) 63(4):232–48. doi: 10.3322/caac.21185 23818335

[22] Erasmus JJ Munden RF . The role of integrated computed tomography positron-emission tomography in esophageal cancer: staging and assessment of therapeutic response. Semin Radiat Oncol (2007) 17(1):29–37. doi: 10.1016/j.semradonc.2006.09.005 17185195

[23] Borggreve AS Goense L van Rossum PSN Heethuis SE van Hillegersberg R Lagendijk JJW . Preoperative prediction of pathologic response to neoadjuvant chemoradiotherapy in patients with esophageal cancer using ^18^F-FDG PET/CT and DW-MRI: A prospective multicenter study. Int J Radiat Oncol Biol Phys (2020) 106(5):998–1009. doi: 10.1016/j.ijrobp.2019.12.038 31987972 PMC7103753

[24] Tsuzuki H Suzuki H Tamaki T Sone M Hanai N . Detection ability of ^18^F-fluorodeoxyglucose positron emission Tomography/Computed tomography for clinical T classification of synchronous esophageal cancer in pharyngeal cancer. Anticancer Res (2022) 42(9):4597–602. doi: 10.21873/anticanres.15963 36039428

[25] Naveed M Kubiliun N . Endoscopic treatment of early-stage esophageal cancer. Curr Oncol Rep (2018) 20(9):71. doi: 10.1007/s11912-018-0713-y 30058019

[26] Lichtenberger JP 3rd Zeman MN Dulberger AR Alqutub S Carter BW Manning MA . Esophageal neoplasms: Radiologic-pathologic correlation. Radiol Clin North Am (2021) 59(2):205–17. doi: 10.1016/j.rcl.2020.11.002 33551082

[27] Groth SS Virnig BA Whitson BA DeFor TE Li ZZ Tuttle TM . Determination of the minimum number of lymph nodes to examine to maximize survival in patients with esophageal carcinoma: data from the surveillance epidemiology and end results database. J Thorac Cardiovasc Surg (2010) 139(3):612–20. doi: 10.1016/j.jtcvs.2009.07.017 19709685

[28] Kleinberg L Brock M Gibson M . Management of locally advanced adenocarcinoma of the esophagus and gastroesophageal junction: Finally a consensus. Curr Treat Options Oncol (2015) 16(7):35. doi: 10.1007/s11864-015-0352-6 26112428 PMC4625398

[29] van Hagen P Hulshof MC van Lanschot JJ Steyerberg EW van Berge Henegouwen MI Wijnhoven BP . Preoperative chemoradiotherapy for esophageal or junctional cancer. N Engl J Med (2012) 366(22):2074–84. doi: 10.1056/NEJMoa1112088 22646630

[30] Wagner AD Grabsch HI Mauer M Marreaud S Caballero C Thuss-Patience P . EORTC-1203-GITCG - the "INNOVATION"-trial: Effect of chemotherapy alone versus chemotherapy plus trastuzumab, versus chemotherapy plus trastuzumab plus pertuzumab, in the perioperative treatment of HER2 positive, gastric and gastroesophageal junction adenocarcinoma on pathologic response rate: a randomized phase II-intergroup trial of the EORTC-gastrointestinal tract cancer group, Korean cancer study group and Dutch upper GI-cancer group. BMC Cancer (2019) 19(1):494. doi: 10.1186/s12885-019-5675-4 31126258 PMC6534855

[31] Fitzgerald TL Efird JT Bellamy N Russo SM Jindal C Mosquera C . Perioperative chemotherapy versus postoperative chemoradiotherapy in patients with resectable gastric/gastroesophageal junction adenocarcinomas: A survival analysis of 5058 patients. Cancer (2017) 123(15):2909–17. doi: 10.1002/cncr.30692 28386965

[32] Lorenzen S Biederstädt A Ronellenfitsch U Reißfelder C Mönig S Wenz F . RACE-trial: neoadjuvant radiochemotherapy versus chemotherapy for patients with locally advanced, potentially resectable adenocarcinoma of the gastroesophageal junction - a randomized phase III joint study of the AIO, ARO and DGAV. BMC Cancer (2020) 20(1):886. doi: 10.1186/s12885-020-07388-x 32933498 PMC7493344

[33] Leichman L Berry BT . Experience with cisplatin in treatment regimens for esophageal cancer. Semin Oncol (1991) 18(1 Suppl 3):64–72. doi: 10.1186/s12885-020-07388-x 2003229

[34] Enzinger PC Kulke MH Clark JW Ryan DP Kim H Earle CC . Phase II trial of irinotecan in patients with previously untreated advanced esophageal and gastric adenocarcinoma. Dig Dis Sci (2005) 50(12):2218–23. doi: 10.1007/s10620-005-3038-2 16416165

[35] Brell JM Krishnamurthi SS Javle M Saltzman J Wollner I Pelley R . A multi-center phase II study of oxaliplatin, irinotecan, and capecitabine in advanced gastric/gastroesophageal junction carcinoma. Cancer Chemother Pharmacol (2009) 63(5):851–7. doi: 10.1007/s00280-008-0807-6 PMC4209292 18670776

[36] Muro K Hamaguchi T Ohtsu A Boku N Chin K Hyodo I . A phase II study of single-agent docetaxel in patients with metastatic esophageal cancer. Ann Oncol (2004) 15(6):955–9. doi: 10.1093/annonc/mdh231 15151954

[37] Ilson DH Wadleigh RG Leichman LP Kelsen DP . Paclitaxel given by a weekly 1-h infusion in advanced esophageal cancer. Ann Oncol (2007) 18(5):898–902. doi: 10.1093/annonc/mdm004 17351256

[38] Ku GY Ilson DH Schwartz LH Capanu M O'Reilly E Shah MA . Phase II trial of sequential paclitaxel and 1 h infusion of bryostatin-1 in patients with advanced esophageal cancer. Cancer Chemother Pharmacol (2008) 62(5):875–80. doi: 10.1007/s00280-008-0677-y 18270704

[39] Harstrick A Bokemeyer C Preusser P Köhne-Wömpner CH Meyer HJ Stahl M . Phase II study of single-agent etoposide in patients with metastatic squamous-cell carcinoma of the esophagus. Cancer Chemother Pharmacol (1992) 29(4):321–2. doi: 10.1007/BF00685952 1537080

[40] Hammel P Huguet F van Laethem JL Goldstein D Glimelius B Artru P . Effect of chemoradiotherapy vs chemotherapy on survival in patients with locally advanced pancreatic cancer controlled after 4 months of gemcitabine with or without erlotinib: The LAP07 randomized clinical trial. JAMA (2016) 315(17):1844–53. doi: 10.1001/jama.2016.4324 27139057

[41] da Costa SCS Bonadio RC Gabrielli FCG Aranha AS Dias Genta MLN Miranda VC . Neoadjuvant chemotherapy with cisplatin and gemcitabine followed by chemoradiation versus chemoradiation for locally advanced cervical cancer: A randomized phase II trial. J Clin Oncol (2019) 37(33):3124–31. doi: 10.1200/JCO.19.00674 31449470

[42] Zhang X Shen L Li J Li Y Li J Jin M . A phase II trial of paclitaxel and cisplatin in patients with advanced squamous-cell carcinoma of the esophagus. Am J Clin Oncol (2008) 31(1):29–33. doi: 10.1097/COC.0b013e3181131ca9 18376224

[43] Ilson DH Ajani J Bhalla K Forastiere A Huang Y Patel P . Phase II trial of paclitaxel, fluorouracil, and cisplatin in patients with advanced carcinoma of the esophagus. J Clin Oncol (1998) 16(5):1826–34. doi: 10.1200/JCO.1998.16.5.1826 9586897

[44] Münch S Pigorsch SU Devečka M Dapper H Weichert W Friess H . Comparison of definite chemoradiation therapy with carboplatin/paclitaxel or cisplatin/5-fluoruracil in patients with squamous cell carcinoma of the esophagus. Radiat Oncol (2018) 13(1):139. doi: 10.1186/s13014-018-1085-z 30068371 PMC6090949

[45] Yano T Wang KK . Photodynamic therapy for gastrointestinal cancer. Photochem Photobiol (2020) 96(3):517–23. doi: 10.1111/php.13206 31886891

[46] Ajani JA D'Amico TA Bentrem DJ Chao J Corvera C Das P . Esophageal and esophagogastric junction cancers, version 2.2019, NCCN clinical practice guidelines in oncology. J Natl Compr Canc Netw (2019) 17(7):855–83. doi: 10.6004/jnccn.2019.0033 31319389

[47] Agostinis P Berg K Cengel KA Foster TH Girotti AW Gollnick SO . Photodynamic therapy of cancer: an update. CA Cancer J Clin (2011) 61(4):250–81. doi: 10.3322/caac.20114 PMC3209659 21617154

[48] Castano AP Mroz P Hamblin MR . Photodynamic therapy and anti-tumour immunity. Nat Rev Cancer (2006) 6(7):535–45. doi: 10.1038/nrc1894 PMC2933780 16794636

[49] Dolmans DE Fukumura D Jain RK . Photodynamic therapy for cancer. Nat Rev Cancer (2003) 3(5):380–7. doi: 10.1038/nrc1071 12724736

[50] Kwiatkowski S Knap B Przystupski D Saczko J Kędzierska E Knap-Czop K . Photodynamic therapy - mechanisms, photosensitizers and combinations. BioMed Pharmacother (2018) 106:1098–107. doi: 10.1016/j.biopha.2018.07.049 30119176

[51] Huang Z . A review of progress in clinical photodynamic therapy. Technol Cancer Res Treat (2005) 4(3):283–93. doi: 10.1177/153303460500400308 PMC1317568 15896084

[52] Greenwald BD . Photodynamic therapy for esophageal cancer. Update. Chest Surg Clin N Am (2000) 10(3):625–37. 10967762

[53] Allison RR Sibata CH . Oncologic photodynamic therapy photosensitizers: a clinical review. Photodiagnosis Photodyn Ther (2010) 7(2):61–75. doi: 10.1016/j.pdpdt.2010.02.001 20510301

[54] Castano AP Demidova TN Hamblin MR . Mechanisms in photodynamic therapy: part one-photosensitizers, photochemistry and cellular localization. Photodiagnosis Photodyn Ther (2004) 1(4):279–93. doi: 10.1016/S1572-1000(05)00007-4 PMC4108220 25048432

[55] Moser JG . Photodynamic tumor therapy 2nd and 3rd generation photosensitizers. Amsterdam: Harwood Academic Publishers (1998).

[56] Juzeniene A Peng Q Moan J . Milestones in the development of photodynamic therapy and fluorescence diagnosis. Photochem Photobiol Sci (2007) 6(12):1234–45. doi: 10.1039/b705461k 18046478

[57] Yano T Hatogai K Morimoto H Yoda Y Kaneko K . Photodynamic therapy for esophageal cancer. Ann Transl Med (2014) 2(3):29. doi: 10.3978/j.issn.2305-5839.2014.03.01 25333005 PMC4200616

[58] Perry Y Epperly MW Fernando HC Klein E Finkelstein S Greenberger JS . Photodynamic therapy induced esophageal stricture - an animal model: from mouse to pig. J Surg Res (2005) 123(1):67–74. doi: 10.1016/j.jss.2004.05.006 15652952

[59] Cramers P Ruevekamp M Oppelaar H Dalesio O Baas P Stewart FA . Foscan uptake and tissue distribution in relation to photodynamic efficacy. Br J Cancer (2003) 88(2):283–90. doi: 10.1038/sj.bjc.6600682 PMC2377038 12610515

[60] Wagnieres G Hadjur C Grosjean P Braichotte D Savary JF Monnier P . Clinical evaluation of the cutaneous phototoxicity of 5,10,15,20-tetra(m-hydroxyphenyl)chlorin. Photochem Photobiol (1998) 68(3):382–7. doi: 10.1111/j.1751-1097.1998.tb09696.x 9747593

[61] Barr H Kendall C Stone N . Photodynamic therapy for esophageal cancer: a useful and realistic option. Technol Cancer Res Treat (2003) 2(1):65–76. doi: 10.1177/153303460300200108 12625755

[62] Bown SG Rogowska AZ . New photosensitizers for photodynamic therapy in gastroenterology. Can J Gastroenterol (1999) 13(5):389–92. doi: 10.1155/1999/454789 10377468

[63] Wu H Minamide T Yano T . Role of photodynamic therapy in the treatment of esophageal cancer. Dig Endosc (2019) 31(5):508–16. doi: 10.1111/den.13353 30667112

[64] Qumseya BJ David W Wolfsen HC . Photodynamic therapy for barrett's esophagus and esophageal carcinoma. Clin Endosc. (2013) 46(1):30–7. doi: 10.5946/ce.2013.46.1.30 PMC3572348 23423151

[65] Sieroń A Kwiatek S . Twenty years of experience with PDD and PDT in Poland - review. Photodiagnosis Photodyn Ther (2009) 6(2):73–8. doi: 10.1016/j.pdpdt.2009.07.003 19683204

[66] . Available at: https://www.accessdata.fda.gov/drugsatfda_docs/label/2011/020451s020lbl.pdf.

[67] Lightdale CJ Heier SK Marcon NE McCaughan JS Jr Gerdes H Overholt BF . Photodynamic therapy with porfimer sodium versus thermal ablation therapy with Nd:YAG laser for palliation of esophageal cancer: a multicenter randomized trial. Gastrointest Endosc. (1995) 42(6):507–12. doi: 10.1016/s00165107(95)70002-1 8674919

[68] Overholt BF Panjehpour M Haydek JM . Photodynamic therapy for barrett's esophagus: follow-up in 100 patients. Gastrointestinal Endoscopy (1999) 49:1–7. doi: 10.1016/S0016-5107(99)70437-2 9869715

[69] Wolfsen HC Woodward TA Raimondo M . Photodynamic therapy for dysplastic Barrett esophagus and early esophageal adenocarcinoma. Mayo Clin Proc (2002) 77(11):1176–81. doi: 10.4065/77.11.1176 12440553

[70] Overholt BF Panjehpour M Halberg DL . Photodynamic therapy for barrett's esophagus with dysplasia and/or early stage carcinoma: long-term results. Gastrointest Endosc (2003) 58(2):183–8. doi: 10.1067/mge.2003.327 12872083

[71] Wolfsen HC Hemminger LL Wallace MB Devault KR . Clinical experience of patients undergoing photodynamic therapy for barrett's dysplasia or cancer. Aliment Pharmacol Ther (2004) 20(10):1125–31. doi: 10.1111/j.1365-2036.2004.02209.x 15569115

[72] Overholt BF Lightdale CJ Wang KK Canto MI Burdick S Haggitt RC . International photodynamic group for high-grade dysplasia in barrett's esophagus. *Photodynamic therapy with porfimer sodium for ablation of high-grade dysplasia in barrett's esophagus: international, partially blinded, randomized phase III trial* . Gastrointest Endosc. (2005) 62(4):488–98. doi: 10.1016/j.gie.2005.06.047 16185958

[73] Overholt BF Wang KK Burdick JS Lightdale CJ Kimmey M Nava HR . International photodynamic group for high-grade dysplasia in barrett's esophagus. *Five-year efficacy and safety of photodynamic therapy with photofrin in barrett's high-grade dysplasia* . Gastrointest Endosc. (2007) 66(3):460–8. doi: 10.1016/j.gie.2006.12.037 17643436

[74] Buttar NS Wang KK Lutzke LS Krishnadath KK Anderson MA . Combined endoscopic mucosal resection and photodynamic therapy for esophageal neoplasia within barrett's esophagus. Gastrointest Endosc (2001) 54(6):682–8. doi: 10.1067/gien.2001.0003 11726842

[75] Prasad GA Wu TT Wigle DA Buttar NS Wongkeesong LM Dunagan KT . Endoscopic and surgical treatment of mucosal (T1a) esophageal adenocarcinoma in barrett's esophagus. Gastroenterology (2009) 137(3):815–23. doi: 10.1053/j.gastro.2009.05.059 PMC3815672 19524578

[76] Yachimski P Puricelli WP Nishioka NS . Patient predictors of histopathologic response after photodynamic therapy of barrett's esophagus with high-grade dysplasia or intramucosal carcinoma. Gastrointest Endosc (2009) 69(2):205–12. doi: 10.1016/j.gie.2008.05.032 18950764

[77] Faybush EM Sampliner RE . Randomized trials in the treatment of barrett's esophagus. Dis Esophagus (2005) 18(5):291–7. doi: 10.1111/j.1442-2050.2005.00503.x 16197527

[78] Peng Q Warloe T Moan J Godal A Apricena F Giercksky KE . Antitumor effect of 5-aminolevulinic acid-mediated photodynamic therapy can be enhanced by the use of a low dose of photofrin in human tumor xenografts. Cancer Res (2001) 61(15):5824–32. 11479222

[79] Gross SA Wolfsen HC . The role of photodynamic therapy in the esophagus. Gastrointest Endosc Clin N Am (2010) 20(1):35–53. doi: 10.1016/j.giec.2009.07.008 19951793

[80] Casas A . Clinical uses of 5-aminolaevulinic acid in photodynamic treatment and photodetection of cancer: A review. Cancer Lett (2020) 490:165–73. doi: 10.1016/j.canlet.2020.06.008 32534172

[81] Dunn J Lovat L . Photodynamic therapy using 5-aminolaevulinic acid for the treatment of dysplasia in barrett's oesophagus. Expert Opin Pharmacother (2008) 9(5):851–8. doi: 10.1517/14656566.9.5.851 18345960

[82] Pech O Gossner L May A Rabenstein T Vieth M Stolte M . Long-term results of photodynamic therapy with 5-aminolevulinic acid for superficial barrett's cancer and high-grade intraepithelial neoplasia. Gastrointest Endosc (2005) 62(1):24–30. doi: 10.1016/s0016-5107(05)00333-0 15990815

[83] Bedwell J MacRobert AJ Phillips D Bown SG . Fluorescence distribution and photodynamic effect of ALA-induced PP IX in the DMH rat colonic tumour model. Br J Cancer (1992) 65(6):818–24. doi: 10.1038/bjc.1992.175 PMC1977757 1616853

[84] Kennedy JC Pottier RH . Endogenous protoporphyrin IX, a clinically useful photosensitizer for photodynamic therapy. J Photochem Photobiol B (1992) 14(4):275–92. doi: 10.1016/1011-1344(92)85108-7 1403373

[85] Peng Q Berg K Moan J Kongshaug M Nesland JM . 5-aminolevulinic acid-based photodynamic therapy: principles and experimental research. Photochem Photobiol (1997) 65(2):235–51. doi: 10.1111/j.17511097.1997.tb08549.x 9066303

[86] Tan WC Fulljames C Stone N Dix AJ Shepherd N Roberts DJ . Photodynamic therapy using 5-aminolaevulinic acid for oesophageal adenocarcinoma associated with barrett's metaplasia. J Photochem Photobiol B (1999) 53(1-3):75–80. doi: 10.1016/s1011-1344(99)00129-3 10672532

[87] Kashtan H Konikoff F Haddad R Skornick Y . Photodynamic therapy of cancer of the esophagus using systemic aminolevulinic acid and a non laser light source: a phase I/II study. Gastrointest Endosc (1999) 49(6):760–4. doi: 10.1016/s0016-5107(99)70297-x 10343224

[88] Godal A Nilsen NO Klaveness J Branden JE Nesland JM Peng Q . New derivatives of 5-aminolevulinic acid for photodynamic therapy: chemical synthesis and porphyrin production in in vitro and in vivo biological systems. J Environ Pathol Toxicol Oncol (2006) 25(1-2):109–26. doi: 10.1615/jenvironpatholtoxicoloncol.v25.i1-2.60 16566712

[89] Andrejevic Blant S Grosjean P Ballini JP Wagnières G van den Bergh H Fontolliet C . Localization of tetra(m-hydroxyphenyl)chlorin (Foscan) in human healthy tissues and squamous cell carcinomas of the upper aero-digestive tract, the esophagus and the bronchi: a fluorescence microscopy study. J Photochem Photobiol B (2001) 61(1-2):1–9. doi: 10.1016/s1011-1344(01)00148-8 11485842

[90] Etienne J Dorme N Bourg-Heckly G Raimbert P Fléjou JF . Photodynamic therapy with green light and m-tetrahydroxyphenyl chlorin for intramucosal adenocarcinoma and high-grade dysplasia in barrett's esophagus. Gastrointest Endosc (2004) 59(7):880–9. doi: 10.1016/s0016-5107(04)01271-4 15173809

[91] Lovat LB Jamieson NF Novelli MR Mosse CA Selvasekar C Mackenzie GD . Photodynamic therapy with m-tetrahydroxyphenyl chlorin for high-grade dysplasia and early cancer in barrett's columnar lined esophagus. Gastrointest Endosc (2005) 62(4):617–23. doi: 10.1016/j.gie.2005.04.043 16185985

[92] Gossner L May A Sroka R Ell C . A new long-range through-the-scope balloon applicator for photodynamic therapy in the esophagus and cardia. Endoscopy (1999) 31(5):370–6. doi: 10.1055/s-1999-31 10433046

[93] Javaid B Watt P Krasner N . Photodynamic therapy (PDT) for oesophageal dysplasia and early carcinoma with mTHPC (m-tetrahydroxyphenyl chlorin): a preliminary study. Lasers Med Sci (2002) 17(1):51–6. doi: 10.1007/s10103-002-8266-5 11845368

[94] Kato H Furukawa K Sato M Okunaka T Kusunoki Y Kawahara M . Phase II clinical study of photodynamic therapy using mono-l-aspartyl chlorin e6 and diode laser for early superficial squamous cell carcinoma of the lung. Lung Cancer (2003) 42(1):103–11. doi: 10.1016/s0169-5002(03)00242-3 14512194

[95] Muragaki Y Akimoto J Maruyama T Iseki H Ikuta S Nitta M . Phase II clinical study on intraoperative photodynamic therapy with talaporfin sodium and semiconductor laser in patients with malignant brain tumors. J Neurosurg (2013) 119(4):845–52. doi: 10.3171/2013.7.JNS13415 23952800

[96] Horimatsu T Muto M Yoda Y Yano T Ezoe Y Miyamoto S . Tissue damage in the canine normal esophagus by photoactivation with talaporfin sodium (laserphyrin): a preclinical study. PloS One (2012) 7(6):e38308. doi: 10.1371/journal.pone.0038308 22719875 PMC3374776

[97] Yano T Muto M Minashi K Ohtsu A Yoshida S . Photodynamic therapy as salvage treatment for local failures after definitive chemoradiotherapy for esophageal cancer. Gastrointest Endosc (2005) 62(1):31–6. doi: 10.1016/s0016-5107(05)00545-6 15990816

[98] Minamide T Yoda Y Hori K Shinmura K Oono Y Ikematsu H . Advantages of salvage photodynamic therapy using talaporfin sodium for local failure after chemoradiotherapy or radiotherapy for esophageal cancer. Surg Endosc (2020) 34(2):899–906. doi: 10.1007/s00464-019-06846-3 31139985

[99] Ishida N Osawa S Miyazu T Kaneko M Tamura S Tani S . Photodynamic therapy using talaporfin sodium for local failure after chemoradiotherapy or radiotherapy for esophageal cancer: A single center experience. J Clin Med (2020) 9(5):1509. doi: 10.3390/jcm9051509 32429571 PMC7290876

[100] Nava HR Allamaneni SS Dougherty TJ Cooper MT Tan W Wilding G . Photodynamic therapy (PDT) using HPPH for the treatment of precancerous lesions associated with barrett's esophagus. Lasers Surg Med (2011) 43(7):705–12. doi: 10.1002/lsm.21112 PMC3218433 22057498

[101] Tanaka T Matono S Nagano T Murata K Sueyoshi S Yamana H . Photodynamic therapy for large superficial squamous cell carcinoma of the esophagus. Gastrointest Endosc (2011) 73(1):1–6. doi: 10.1016/j.gie.2010.08.049 21074765

[102] Yano T Muto M Minashi K Iwasaki J Kojima T Fuse N . Photodynamic therapy as salvage treatment for local failure after chemoradiotherapy in patients with esophageal squamous cell carcinoma: a phase II study. Int J Cancer (2012) 131(5):1228–34. doi: 10.1002/ijc.27320 22024814

[103] Yano T Muto M Yoshimura K Niimi M Ezoe Y Yoda Y . Phase I study of photodynamic therapy using talaporfin sodium and diode laser for local failure after chemoradiotherapy for esophageal cancer. Radiat Oncol (2012) 7:113. doi: 10.1186/1748-717X-7-113 22824179 PMC3410784

[104] Lindenmann J Matzi V Neuboeck N Anegg U Baumgartner E Maier A . Individualized, multimodal palliative treatment of inoperable esophageal cancer: clinical impact of photodynamic therapy resulting in prolonged survival. Lasers Surg Med (2012) 44(3):189–98. doi: 10.1002/lsm.22006 22334351

[105] Yano T Kasai H Horimatsu T Yoshimura K Teramukai S Morita S . A multicenter phase II study of salvage photodynamic therapy using talaporfin sodium (ME2906) and a diode laser (PNL6405EPG) for local failure after chemoradiotherapy or radiotherapy for esophageal cancer. Oncotarget (2017) 8(13):22135–44. doi: 10.18632/oncotarget.14029 PMC5400653 28212527

[106] Foroulis CN Thorpe JA . Photodynamic therapy (PDT) in barrett's esophagus with dysplasia or early cancer. Eur J Cardiothorac Surg (2006) 29(1):30–4. doi: 10.1016/j.ejcts.2005.10.033 16337389

[107] Gray J Fullarton GM . Long term efficacy of photodynamic therapy (PDT) as an ablative therapy of high grade dysplasia in barrett's oesophagus. Photodiagnosis Photodyn Ther (2013) 10(4):561–5. doi: 10.1016/j.pdpdt.2013.06.002 24284112

[108] Dunn JM Mackenzie GD Banks MR Mosse CA Haidry R Green S . A randomised controlled trial of ALA vs. photofrin photodynamic therapy for high-grade dysplasia arising in barrett's oesophagus. Lasers Med Sci (2013) 28(3):707–15. doi: 10.1007/s10103-012-1132-1 22699800

[109] Pacifico RJ Wang KK Wongkeesong LM Buttar NS Lutzke LS . Combined endoscopic mucosal resection and photodynamic therapy versus esophagectomy for management of early adenocarcinoma in barrett's esophagus. Clin Gastroenterol Hepatol (2003) 1(4):252–7. doi: 10.1016/S1542-3565(03)00129-0 15017665

[110] Ertan A Zaheer I Correa AM Thosani N Blackmon SH . Photodynamic therapy vs radiofrequency ablation for barrett's dysplasia: efficacy, safety and cost-comparison. World J Gastroenterol (2013) 19(41):7106–13. doi: 10.3748/wjg.v19.i41.7106 PMC3819546 24222954

[111] Davila ML . Photodynamic therapy. Gastrointest Endosc Clin N Am (2011) 21(1):67–79. doi: 10.1016/j.giec.2010.09.002 21112498

[112] Ebigbo A Karstensen JG Aabakken L Dinis-Ribeiro M Spaander M Le Moine O . Esophageal stenting for benign and malignant disease: European society of gastrointestinal endoscopy (ESGE) cascade guideline. Endosc Int Open (2019) 7(6):E833–6. doi: 10.1055/a-0898-3523 PMC6561763 31198848

[113] Roque JA 3rd Barrett PC Cole HD Lifshits LM Bradner E Shi G . Os(II) oligothienyl complexes as a hypoxia-active photosensitizer class for photodynamic therapy. Inorg Chem (2020) 59(22):16341–60. doi: 10.1021/acs.inorgchem.0c02137 PMC7669743 33126792

[114] Alsaab HO Alghamdi MS Alotaibi AS Alzhrani R Alwuthaynani F Althobaiti YS . Progress in clinical trials of photodynamic therapy for solid tumors and the role of nanomedicine. Cancers (Basel) (2020) 12(10):2793. doi: 10.3390/cancers12102793 33003374 PMC7601252

[115] Palazzolo S Bayda S Hadla M Caligiuri I Corona G Toffoli G . The clinical translation of organic nanomaterials for cancer therapy: A focus on polymeric nanoparticles, micelles, liposomes and exosomes. Curr Med Chem (2018) 25(34):4224–68. doi: 10.2174/0929867324666170830113755 28875844

[116] Didamson OC Abrahamse H . Targeted photodynamic diagnosis and therapy for esophageal cancer: Potential role of functionalized nanomedicine. Pharmaceutics (2021) 13(11):1943. doi: 10.3390/pharmaceutics13111943 34834358 PMC8625244

[117] Zhao W Zhao J Kang L Li C Xu Z Li J . Fluoroscopy-guided salvage photodynamic therapy combined with nanoparticle albumin-bound paclitaxel for locally advanced esophageal cancer after chemoradiotherapy: A case report and literature review. Cancer Biother Radiopharm. (2022) 37(5):410–6. doi: 10.1089/cbr.2020.4595 33794100

[118] Aebisher D Zamadar M Mahendran A Ghosh G McEntee C Greer A . Fiber-optic singlet oxygen [^1^O_2_ (^1^Δg)] generator device serving as a point selective sterilizer. Photochem Photobiology. (2010) 86:890–4. doi: 10.1111/j.1751-1097.2010.00748.x PMC4845035 20497367

[119] Aebisher D Azar N Zamadar M Gandra N Gafney H Gao R . Singlet oxygen chemistry in water. a porous vycor glass supported photosensitizer. J Phys Chem B (2008) 112(7):1913–7. doi: 10.1111/j.1751-1097.2010.00748 18225891

[120] Moan J Berg K . The photodegradation of porphyrins in cells can be used to estimate the lifetime of singlet oxygen. Photochem Photobiol (1991) 53:549. doi: 10.1111/j.1751-1097.1991.tb03669.x 1830395

[121] Niedre M Patterson MS Wilson BC . Direct near-infrared luminescence detection of singlet oxygen generated by photodynamic therapy in cells *in vitro* and tissues *in vivo photochem* . Photobiol (2002) 75:392. doi: 10.1562/0031-8655(2002)0750382DNILDO2.0.CO2 12003128

[122] Chen J Stefflova K Niedre MJ Wilson BC Chance B Glickson JD . Protease-triggered photosensitizing beacon based on singlet oxygen quenching and activation. J Am Chem Soc (2004) 126:11450. doi: 10.1021/ja047392k 15366886

