# Supplementary material for: Advancements in photodynamic therapy of esophageal cancer
Source: Front Oncol. 2022 Nov 17;12:1024576. doi: 10.3389/fonc.2022.1024576 (PMC9713848; doi:10.3389/fonc.2022.1024576)
Supplement: Supplementary file 1 [file DataSheet_1.docx]

~~
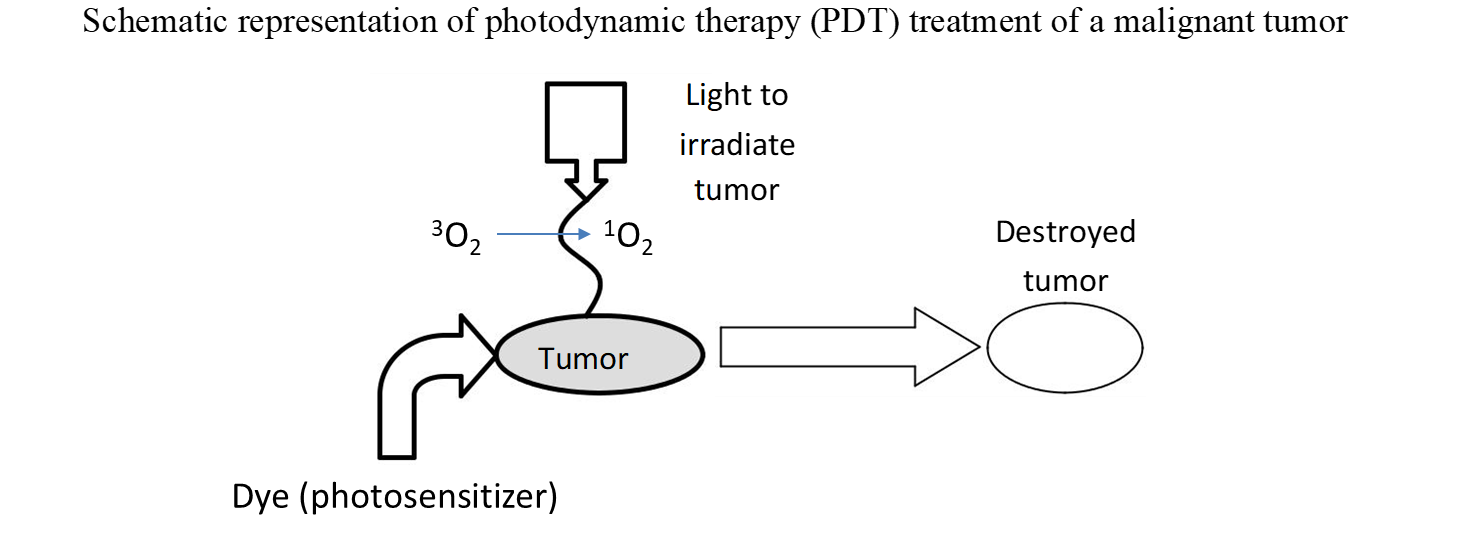
~~

Supplementary Figure 1. PDT treatment of tumor


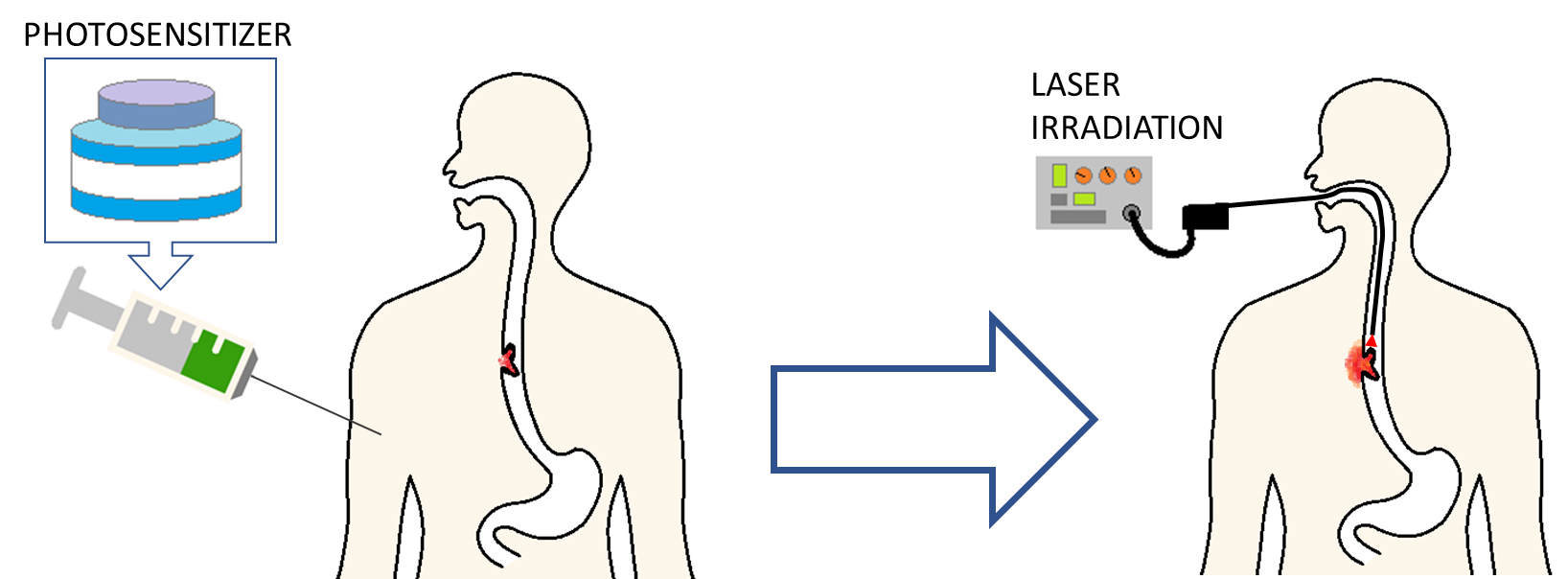


Supplementary Figure 2. PDT procedure


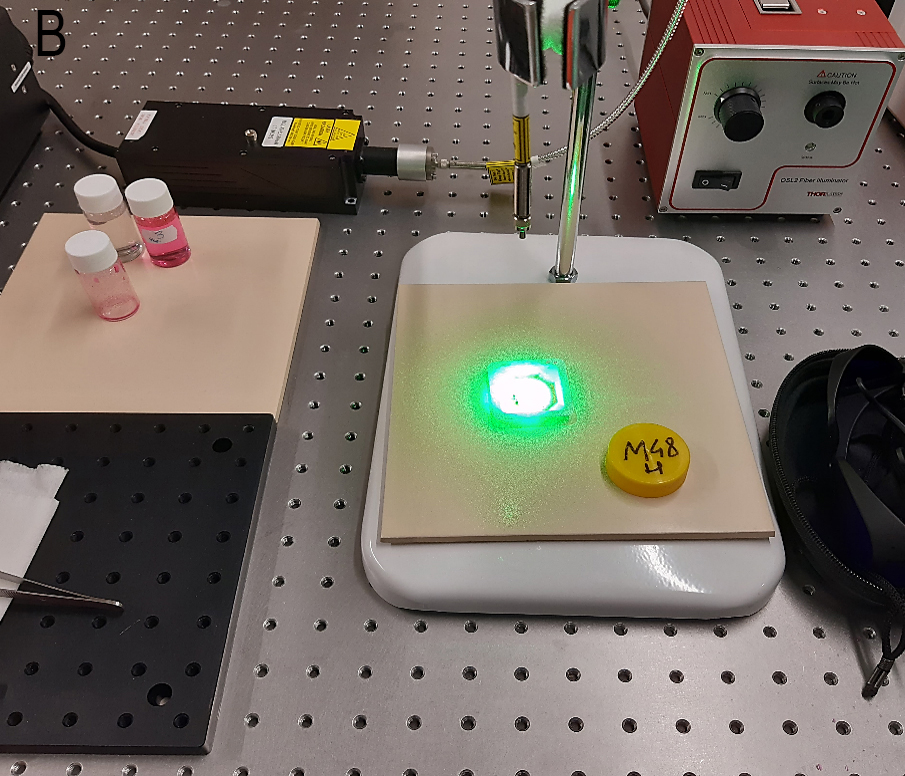


Supplementary Figure 3. Experimental setup during irradiation of sample (own photo).


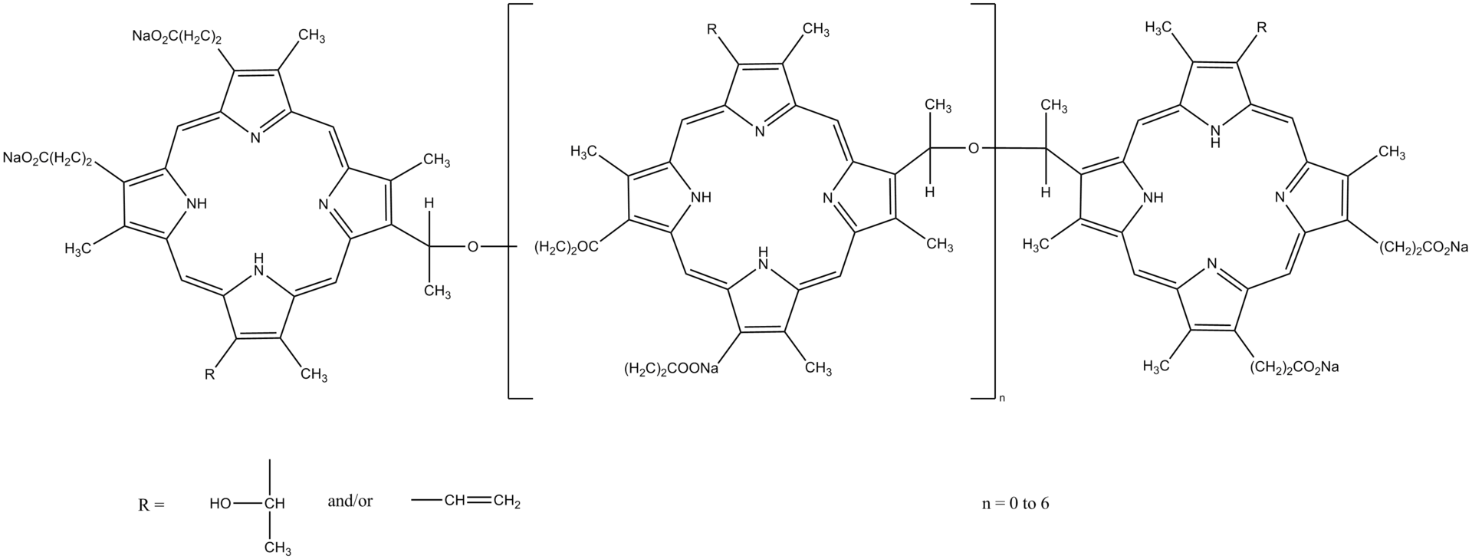
 Supplementary Figure 4. The structure of Porfimer sodium


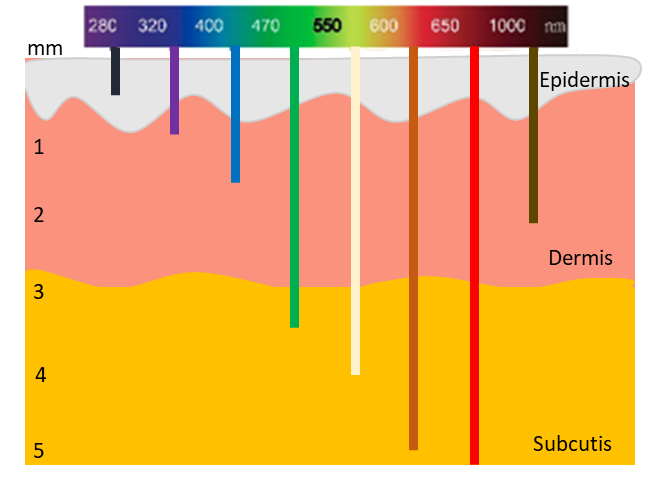


Supplementary Figure 5. PDT action localized in tissue, in regards of applied wavelength.


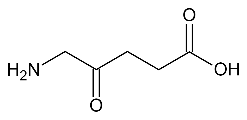


Supplementary Figure 6. The structure of 5-ALA.


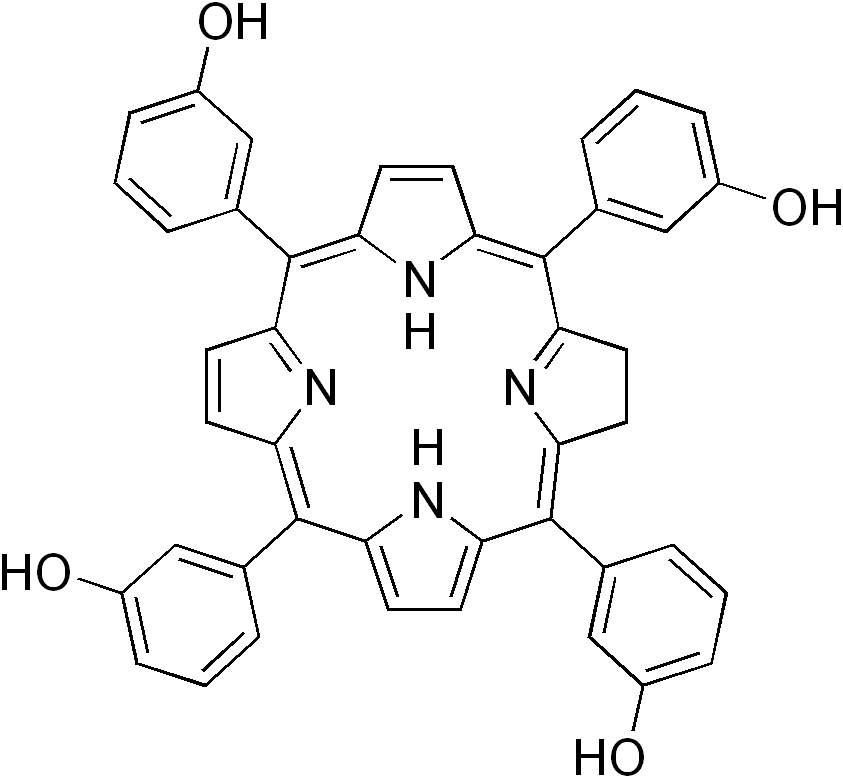


Supplementary Figure 7. The structure of mTHPC.


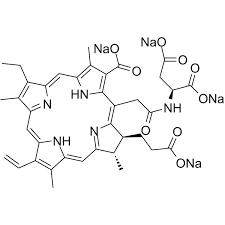


Supplementary Figure 8. Talaporfin sodium.


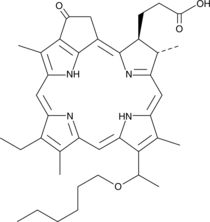


Supplementary Figure 9. 2-[1-hexyloxyethyl]-2-devinyl pyropheophorbide-a

**
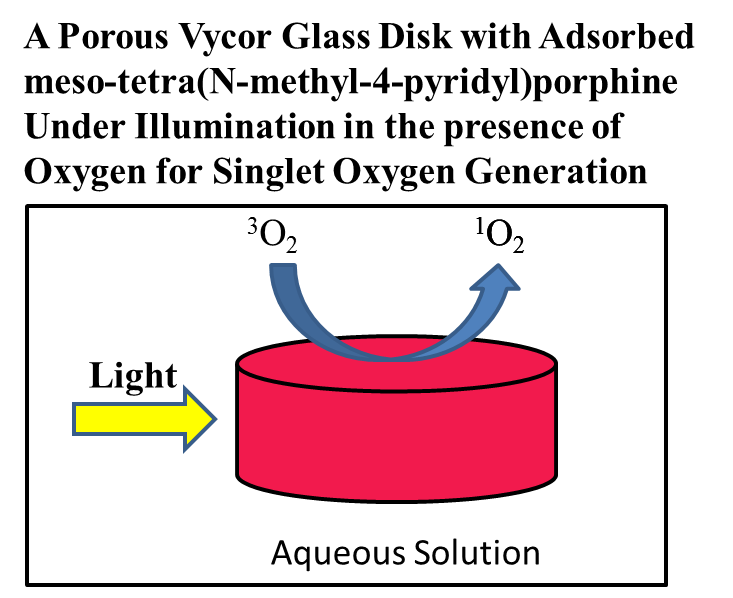
**

Supplementary Figure 10. Solid support for PS
